# UV cross-linked polyvinylpyrrolidone electrospun fibres as antibacterial surfaces

**DOI:** 10.1080/14686996.2019.1667737

**Published:** 2019-09-17

**Authors:** Barbara M. Maciejewska, Jacek K. Wychowaniec, Marta Woźniak-Budych, Łukasz Popenda, Alicja Warowicka, Klaudia Golba, Jagoda Litowczenko, Zbigniew Fojud, Beata Wereszczyńska, Stefan Jurga

**Affiliations:** a NanoBioMedical Centre, Adam Mickiewicz University, Poznań, Poland; b Department of Animal Physiology and Development, Faculty of Biology, Adam Mickiewicz University, Poznań, Poland; c Department of Molecular Virology, Faculty of Biology, Adam Mickiewicz University, Poznań, Poland; d Department of Macromolecular Physics, Faculty of Physics, Adam Mickiewicz University, Poznań, Poland

**Keywords:** Crosslinking, electrospinning, polymer fibres, lysozyme, antibacterial, benzophenone, polyvinylpyrrolidone, 103 Composites, 212 Surface and interfaces, 306 Thin film / Coatings, 503 TEM, STEM, SEM, 501 Chemical analyses, 505 Optical / Molecular spectroscopy

## Abstract

Many bacteria become progressively more resistant to antibiotics and it remains a challenging task to control their overall levels. Polymers combined with active biomolecules come to the forefront for the design of antibacterial materials that can address this encounter. In this work, we investigated the photo-crosslinking approach of UV-sensitive benzophenone molecule (BP) with polyvinylpyrrolidone (PVP) polymer within electrospun fibres. The BP and PVP solutions allowed fabricating polymer mats that were subsequently functionalised with antibacterial lysozyme. The physical properties of the crosslinked electrospun fibres were investigated by scanning electron microscopy and atomic force microscopy. The average diameter of the obtained fibres decreased from 290 ± 50 nm to 270 ± 70 nm upon the addition of the crosslinking molecules and then to 240 ± 80 nm and 180 ± 90 nm after subsequent crosslinking reaction at an increasing time: 3 and 5 h, respectively. The peak force quantitative nanomechanical mapping (PF-QNM) indicated the increase of DMT modulus of obtained cross-linked fibres from 4.1 ± 0.8 GPa to 7.2 ± 0.5 GPa. Furthermore, the successful crosslinking reaction of PVP and BP solution into hydrogels was investigated in terms of examining photo-crosslinking mechanism and was confirmed by rheology, Raman, Fourier transform infrared and nuclear magnetic resonance. Finally, lysozyme was successfully encapsulated within cross-linked PVP-BP hydrogels and these were successfully electrospun into mats which were found to be as effective antibacterial agents as pure lysozyme molecules. The dissolution rate of photo cross-linked PVP mats was observed to increase in comparison to pure PVP electrospun mats which opened a potential route for their use as antibacterial, on-demand, dissolvable coatings for various biomedical applications.

## Graphical Abstract

**Figure:**
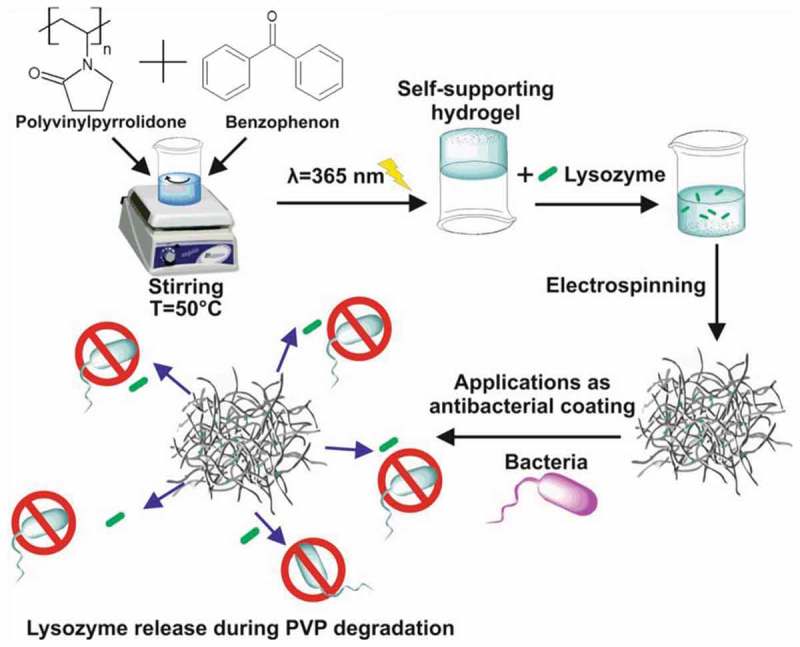

## 1. Introduction

Bacteria attachment and colonisation to the surfaces usually leads to biofilm formation and causes problematic issues in human healthcare and industrial applications [1]. Preserving the sterility of the surfaces is especially important in hospital facilities including surgical equipment, but is not limited to water purification systems, packaging materials as well as public health places [2]. The prevention from the adhesion of microorganisms on the publicly used surfaces is a challenge due to increasing resistance of bacteria to antibiotics often causing serious life threats [3]. Staphylococcus aureus (*S. aureus*) and methicillin-resistant *Staphylococcus aureus* (MRSA) cause a high percentage of hospital-acquired infections [4]. Therefore, the fabrication of antimicrobial coatings of appropriate physicochemical properties remains important. In particular, surfaces and coatings decorated with antimicrobial molecules or active nanoparticles became a key strategy for the bacterial-preventive materials [5–9]. The ideal antibacterial surface should fulfil several requirements, specifically (i) prevent initial bacterial attachment and biofilm formation, (ii) kill bacteria that overcome the barrier (if they do), and (iii) remove dead bacteria or/and bacterial debris and toxins from the coating [10]. The economic burden also dictates that they should be cost-effective, scalable and biodegradable [11]. Bactericidal surfaces, i.e. coatings possessing ability to kill bacteria and/or inhibit their growth can be divided into two major groups. The first one, the contact-based bactericidal coatings have attracted significant scientific interest due to their easiness of functionalisation by active (bio) molecules covalent bonding or by physical adsorption as well as easy antibacterial action by molecule-bacteria contact. The second one, called the release-based coating, contains biocides preloaded or embedded within their structure, which are released slowly and/or in a controllable manner specifically to damage bacteria when necessary [10].

Polymeric fibres become the leading antimicrobial materials due to their easiness of fabrication into well-defined layered structures and potential for antimicrobial molecules encapsulation due to the possibility of physical and chemical functionalisation [12,13]. Such fibrous coatings can be fabricated via electrospinning [14]. This approach in which fine, polymer-based fibres are formed from a liquid suspension, using the electrostatic forces is already a well-established method [15]. Such controlled synthesis of polymeric fibrous nanomaterials paves the way to a special class of biomaterials with the unique tunable structure, and physicochemical and antibacterial properties [16]. De Faria et al. demonstrated that poly lactic-co-glycolic acid (PLGA)-chitosan fibrous mats functionalised with graphene oxide-silver (GO-Ag) nanocomposites were able to inactivate both Gram-positive and Gram-negative bacteria, however, the procedure of their preparation was time-consuming [17]. The hybrid efficient bioresponsive systems with controlled release of antibacterial activity via metal ion coordination polymer on titanium nanotubes were proved to be controllable delivery system for the long-lasting treatment of biomaterial-related bacterial infections [18]. Another example of materials which can constitute the base for systems with antibacterial activity is polycaprolactone, remaining one of the most commonly used polymer for biomedical applications often used in fibrous mats production via electrospinning method [19]. Polymers which can be used to fabricate electrospun fibrous mats and possess antimicrobial properties are of particular interest to scientists since they do not require exogenous supply of active antimicrobial biomolecules. Their typical action is due to excessive cationic surface charge that can affect the integrity of bacterial cell wall [20–22].

In case of some hydrophilic polymers which can be easily dispersed in water, additional crosslinking procedure should be performed to decrease the rate of solubility, make the polymer chain network integrated and overall prolong the coating’s degradation time [23]. A polymer with an integrated network can be obtained by a crosslinking process, in which the polymer chains are connected by intra- or intermolecular covalent or non-covalent links [24]. Several crosslinking approaches including the temperature, radiation, external reactants and processing are commonly used to induce/generate the covalent or physical crosslinking as well as ionic bonding [25]. Frequently additional molecules which enhance and/or initiate that process are used [26]. The widely studied, due to its ease of use, high photochemical activity and low price, is a benzophenone (BP) molecule. BP is a photoinitiator which after the absorption of UV quanta excites to a singlet state that immediately goes the intersystem crossing to return to the lower energy reactive triplet [27]. This ability of BP to form covalent bonding with copolymers and greatly influence the gelation efficiency due to radical generation on the polymer backbone after UV irradiation was already exploited in the literature [28]. Benzophenone and its derivatives were also found to have significant antibacterial activity [29–31]. There are number of synthetic biocides that inhibit the growth of bacteria [32,33], however, the substances of natural origin secreted by living organisms including hydrolytic enzymes which act more specifically, are considered as more attractive class of biocidal agents due to their biological nature [34]. One of such popular hydrolytic enzymes is lysozyme, a powerful antibacterial enzyme which breaks the chemical bonds in the outer cell wall of bacteria, leading to its damage and eventual death [35]. Reports indicate that lysozyme acts more effectively for Gram-positive bacteria [36]. Beside antimicrobial activity, lysozyme has many other functions, including inactivation of certain viruses [37]. It was also observed that lysozyme molecule participates in removing bacterial cell walls after killing the bacteria on the surface where the molecule was applied. Lysozyme’s well-known antibacterial properties in fact have been further investigated by a number of scientists who incorporated it into/on surfaces to form antimicrobial coatings. Yuan et al. introduced the way of antibacterial thin coating formation on stainless steel which was based on polyethylene glycol coupled with lysozyme and showed that such coating reduces the bacterial adhesion and biofilm formation [38]. Pluronic triblock copolymer conjugated with lysozyme enzyme was also proved to act as antiadhesive and antibacterial coating [39], overall indicating the potential of antibacterial lysozyme encapsulated in surfaces.

In this work, our efforts focused on producing antimicrobial mats based on UV-cross-linked polyvinylpyrrolidone (PVP-BP) polymer fibres functionalised with egg lysozyme (rhodamine B labelled). Firstly, the rheological properties of PVP aqueous solutions were investigated to establish the key viscosity parameters required for successful electrospinning. Then, the fibrillar mats were fabricated using in-house electrospinning system. Due to the poor stability of PVP mats in the humid environment, we investigated two UV crosslinking approaches of (1) PVP and BP solutions which were subsequently electrospun and photo-crosslinked and (2) photo-crosslinked PVP and BP solutions including attempt to spin the fibre from crosslinked hydrogel. The effect of UV exposure on the physicochemical properties of BP cross-linked PVP hydrogels and mats/fibres was examined using scanning electron microscopy, atomic force microscopy, Raman spectroscopy, Fourier transform infrared spectroscopy, and nuclear magnetic resonance spectroscopy. The successful incorporation of rhodamine B labelled lysozyme molecules was confirmed by confocal microscopy. Finally, the antimicrobial properties of the mats made of pure PVP and cross-linked PVP functionalised with lysozyme were tested on *S. aureus* bacterial strains using atomic force microscopy. Their effectiveness was demonstrated by observing the damage of bacterial cell walls by the atomic force microscopy images. Moreover, we analysed the cytotoxicity of obtained mats using fibroblasts cells.

## 2. Materials and methods

### 2.1. Preparation of PVP mats

PVP with an average molecular weight of 360 000 was purchased from Sigma Aldrich (Germany). 13%, 15% or 17% w/w PVP was dissolved in deionised (DI) water (with R = 19 Ω) under magnetic stirring at room temperature (RT = 21°C). Fibrous mats from PVP were prepared using the homemade electrospinning system, described in detail in our previous work [40]. The PVP solution was placed in 1 mL syringe with the mean diameter of 0.5 mm and pulled out by a syringe pump. PVP electrospun mats were prepared using flow rate of 2 mL/h, accelerating voltage of 10 kV, at T = 25°C and chamber humidity 30%<H < 37% and a distance between needle and collector of 14 cm.

### 2.2. Preparation of cross-linked PVP mats by means of benzophenone

The amount of 12.0 ± 0.5 mg (per 1 mL of PVP solution) of crosslinking agent: benzophenone (BP) (Sigma Aldrich) was dispersed in 10 µL of ethanol (≥99.5%). Such highly concentrated BP ethanol solution was carefully placed inside the aqueous 17% w/w PVP solution at 50°C to obtain final BP concentration of 2% w/w (PVP-BP). This mixture was left for 30 min at 50°C under vigorous magnetic stirring to disperse the BP molecules homogenously. Such prepared solutions were cooled down to room temperature prior to electrospinning. The same parameters (height, humidity, RT, flow rate and voltage) as above were used for electrospinning of PVP-BP. To crosslink the BP molecules within the produced fibres, produced mats were irradiated with a UV light (λ = 365 nm), for 3, 5 or 10 h in air.

### 2.3. Incorporation of lysozyme to produced mats

Two methods were used to incorporate rhodamine-labelled lysozyme (BIOLIM) into the produced mats. In the first method, PVP-BP solution as described above was irradiated with UV light (λ = 365 nm, P = 24 W) for 3 h to induce crosslinking prior to electrospinning. This process led to formation of a self-supporting, chemically partially crosslinked hydrogel. Subsequently, 0.01 g of lysozyme was mixed into the hydrogel at T = 25°C under vigorous stirring to form PVP-BP-LY hydrogel with 0.25% w/w of lysozyme. This sample was then electrospun using increased flow rate of 5 mL/h and the distance between needle and collector of 8 cm, whilst other electrospinning parameters remained as abovementioned. Such produced mats were consecutively named PVP-BP-LY 3hUV throughout the manuscript.

### 2.4. Scanning electron microscopy (SEM)

The morphology and structure of PVP based fibre s was studied under a Scanning Electron Microscope (SEM Jeol 7001TTLS) with the accelerating voltage between 2 and 4 kV. Prior to measurements, all fibrous mats were placed on the Si substrate and these were subsequently covered by a thin layer of gold (Au) using sputtering method.

### 2.5. Raman spectroscopy

Raman spectroscopy data were obtained on an In Via Renishaw confocal Raman microscope equipped with a 50×microscope objective (LEICA) of numerical aperture n = 0.75, and λ = 785 nm He/Ne laser (with laser power P = 1.5 mW) and a 1800 gr mm^−1^ grating. BP and PVP were measured in a powder form, PVP fibrous mats were measured as-produced from electrospinning and lysozyme was measured in a powder form. Each Raman spectrum was obtained in the range of 1000 to 2500 cm^–1^ corrected by the WiRE^TM^ 3.3 software.

### 2.6. Atomic force microscopy (AFM)

AFM measurements were performed using the Icon Scanning Probe Microscope (Bruker, USA). PVP fibres were electrospun according to above protocol for 30 s to obtain single fibres covering the SiO_2_ substrate. Samples were scanned in air, at RT using tapping mode with n-type antimony-doped Si (RTESPA-300, f: 300 kHz, k = 40 N/m) tips (Bruker, USA). The images were acquired at 512 × 512 pixels resolution over 20 × 20 µm areas at a scan rate of 0.3 Hz. All images were second order flatten using Nanoscope software. The RTESPA tips with higher spring constants of 80 N/m were used in air in the peak force quantitative nanomechanical (PF-QNM) mode on the individual fibres to obtain Derjaguin, Muller and Toporov model (DMT) modulus (Young’s modulus) using following equation:

(1) $$E_{r}=\frac{3\left(F_{tip}-F_{adh}\right)}{4\sqrt{Rd^{3}}}$$

where $E_{r}$ is the reduced Young’s modulus, $F_{tip}$ is the force on the tip, $F_{adh}$ is the adhesion force, and R and d are AFM tip radius and deformation depth, respectively. The AFM tip radius was estimated by normalizing the DMT values to these of calibration polystyrene Film (PS, $E_{r}=2.7GPa$, Bruker, USA). The DMT values of fibres were normalised to the SiO_2_ substrate. The PF-QNM maps were acquired at 512 × 512 pixels resolution over 10 × 10 µm^2^ areas at a scan rate of 1 Hz.

Investigations of bacterial structures were performed in a scan-assist mode using scan-assist fluid tips (f: 150 kHz, k = 0.7 N/m, Bruker, USA). Freshly cleaved mica was incubated with a 10 µL (1% w/v) solution of poly-L-lysine (Sigma Aldrich) for 2 min followed by rinsing with 1 mL of ultra-purified water and drying in air. Then, 10 µL bacteria incubated for 24 h with all required samples (see further method below) were carefully placed onto the poly-L-lysine covered mica for 2 min and again followed by rinsing with 1 mL of ultra-purified water and drying in air prior to AFM imaging. These images were acquired at 512 × 512 pixels resolution over different range of areas (10 x 10 µm^2^, 20 × 20 µm^2^ and 70 × 70 µm^2^) at a scan rate of 0.5 Hz. Some images were second order flattened using Nanoscope software to correct for drifts during scanning.

### 2.7. Rheology

The rheological measurements were performed on a RheoStress RS150 Haake rheometer using cone-plate arrangement with a cone of 35 mm diameter and 1° angle. Viscous polymer solutions and/or hydrogels were carefully placed on the bottom plate using spatula and subsequently, the top rheometer cone-plate was slowly lowered to the desired gap to minimise sample disruption. Solutions viscosity as a function of the shear rate was measured in a steady shear, with shear rates from 1 to 100 s^–1^. Frequency sweeps were performed from 0.1 to 50 Hz at 1% strain within the linear viscoelastic regime of the samples. All measurements were repeated at least three times to ensure reproducibility. All rheological measurements were performed at RT.

### 2.8. Nuclear magnetic resonance (NMR) analysis

NMR spectra were recorded by means of NMR Agilent DD2 800 MHz system in 60% H_2_O/40% D_2_O, using trimethylsilylpropanoic acid (TSP) as the internal standard (δ_H_ = 0.0 ppm, δ_C_ = 0.0 ppm). All spectra were measured at temperature T = 25°C unless otherwise stated.

### 2.9. Fourier transform infrared spectroscopy (FTIR)

Measurements were performed on a Bruker TENSOR 27 FTIR spectrometer equipped with GLOBAR source, KBr beamsplitter, single reflection diamond Platinum ATR accessory and a mercury cadmium telluride detector. Spectra in the 400 to 4000 cm^−1^ range were obtained using resolution of 2 cm^−1^ at T = 25°C and were averaged over 256 scans to ensure a good signal-to-noise ratio. FTIR spectra were transformed using the third order of Blackman–Harris apodisation. The baseline correction of the fifth degree polynomial was applied using Opus 7.5 software (Bruker). All spectra were normalised to the peak at 1634 cm^−1^. An air background was subtracted from all spectra. Measurements were done in triplicate to ensure reproducibility.

### 2.10. Bacteriological tests

The antibacterial activity of all materials was evaluated against *Staphylococcus aureus* (*S. aureus*) bacteria strain. Bacteria were purchased from Polish collection of Microorganisms, Ludwik Hirszfeld Institute of Immunology and Experimental Therapy, Polish Academy of Sciences, Wroclaw. The *S. aureus* were incubated at T = 37°C in a lysogeny broth (LB) medium to an approximate concentration of 10^7^ colony (CFU/mL). Next, to each vial with the proper amount of *S. aureus*, PVP, PVP-BP, PVP-BP cross-linked for 3 h (PVP-BP-LY 3hUV) and Lysozyme were added and cultured for 24 h, respectively. The negative control sample was *S. aureus* culture cultivated in pure LB medium. The positive control sample was *S. aureus* culture cultivated with antibiotic ampicillin.

### 2.11. Cell culture

The human immortalised fibroblasts cell line (MSU-1.1) was obtained through the courtesy of Prof C Kieda (CBM, CNRS, Orleans, France). Cell culture was maintained in sterile cell culture flasks with Dulbecco’s modified Eagle’s culture medium (DMEM, Gibco) supplemented with 10% fetal bovine serum (FBS, Sigma-Aldrich), 100 U/mL penicillin and 100 µg/mL streptomycin (Sigma-Aldrich). Cells were cultured at T = 37°C in a humidified atmosphere of 5% CO_2_. After reaching about 80% confluency the medium was removed and the cells were washed with Hank’s Balanced Salt Solution (HBSS, Sigma-Aldrich) and subcultured by trypsinisation in 1% Trypsin-EDTA solution in PBS (trypsin-ethylenediaminetetraacetic acid, Sigma-Aldrich). The cells were counted using TC10TM automated cell counter (BioRad).

### 2.12. Viability test

The quantitative determination of cell viability after their culture for 24, 48, 72 h with PVP mat, PVP-BP, PVP-BP cross-linked for 3 h (PVP-BP 3 h) and BP was performed using the colorimetric WST-1 assay (Clontech, Takara). The assay is based on the ability of mitochondrial dehydrogenase to reduction of WST-1 to a coloured (dark yellow) formazan product in live cells. Two controls: positive (10% dimethyl sulfoxide in DMEM) and negative (cells seeded in empty non-treated plastic wells containing DMEM) were used. Moreover, cell viability was assessed after incubation of MSU-1.1 with BP. Cells were seeded on PVP, PVP-BP and PVP-BP 3 h mats in the sterile 96-well plates at a density of 1 × 10^4^ cells/well and incubated overnight at T = 37°C in a humidified environment. All materials were sterilised prior to cell culture using UV irradiation (λ = 254 nm) for 30 min on each side. After 24, 48 and 72 h of incubation, 10 µL of WST-1 reagent was added to each well and incubated for an additional 2 h at 37°C. Absorbance of all samples was then measured at λ = 450 nm using a spectrophotometer (Biochrom Anthos Zenyth 340 Microplate Reader). All experiments were repeated in triplicate (n = 3).

## 3. Results and discussion

**Figure 1.**
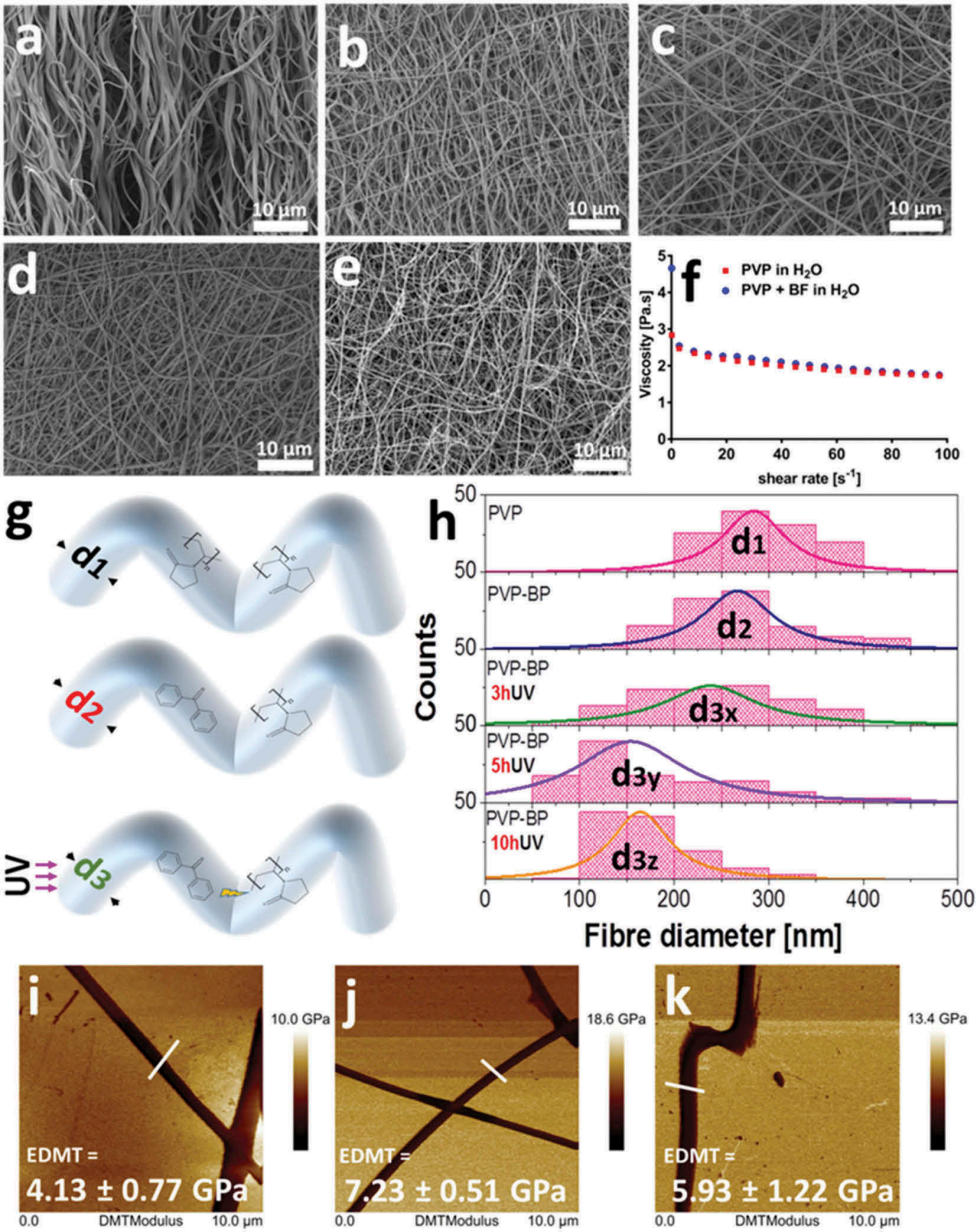
SEM micrographs of: PVP based fibre mat without (a) and with 2% w/w BP after 0 (b), 3 (c), 5 (d) and 10 h (e) of UV exposure. Viscosity as a function of shear rate of the 17% PVP solution in comparison to 17% w/w PVP solution with encapsulated (but not under any UV exposure) 2% w/w BP (f). Histograms of PVP fibre diameters; control PVP, 2% w/w BP, 2% w/w BP after 3, 5 and 10 h of UV exposure (g, h). Diameters were extracted from at least 200 fibres measured individually from a series of randomly chosen SEM images using ImageJ software. PF-QNM AFM DMT Modulus images of individual PVP fibres (i) control (j), 2% w/w BP after 3 h and (k), 5 h of UV exposure. The average values presented in the graph (± SD) are taken over the selection of all points along at least three fibres (n = 3) and referenced to the value of Si substrate, here taken as 0.

In the first step, we have prepared the aqueous suspensions of PVP at the three incrementally increased concentrations: 13%, 15% and 17% w/w. The similar morphology of electrospun mats from all three concentrations was observed under SEM (Figure ESI 1). We observed that only mats produced from 17% w/w aqueous PVP had acceptable reproducibility, with mats formed by smooth fibres (Figure 1(a)) of well-defined size distribution with an average fibre size of 290 ± 50 nm (Figure 1(g,h)). Indeed, the rheological measurements indicated that 17% w/w PVP solution (Figure 1(f)) has sufficiently high viscosity (*η*) at the shear rates that match flow rates used during electrospinning process, and agrees with values reported by others [41]. Furthermore, it can be seen that the 17% PVP solution exhibits shear-thinning behaviour in the used shear rate regime (1–100 s^–1^), which is a critical condition for higher-viscosity solutions to be electrospun [42]. It is well known, that upon water immersion, the electrospun mats produced from 17% w/w PVP solution, completely solubilise within minutes [43]. Therefore, we decided to stabilize formed PVP fibres by means of photo-crosslinking. To do so, we used a standard crosslinking molecule from the diarylketones family: a benzophenone [44]. The ability of BP molecules to become cross-linkers under UV light (λ = 365 nm) [28] and their low melting temperature of 48.5°C [45], present a facile route for the preparation of crosslinked PVP mats more stable in aqueous environments for longer period of time than that of pure PVP electrospun fibres. Therefore, we have then dispersed highly concentrated (1.2 mg/µL) benzophenone (BP) in aqueous PVP solution (PVP-BP) at 50°C to obtain the final BP concentration of 2% w/w. Shear-rheology experiments showed no significant difference between viscosity values of aqueous PVP solution and PVP-BP (Figure 1(f)). The solutions of PVP-BP were then electrospun to fabricate a selection of fibrous mats. The diameter distribution of the fibres was very similar to that of pure PVP fibres (Figure 1(a,b,g,h)) with an average diameter size 270 ± 70 nm. These polymer mats were then subjected to 3, 5 or 10-h UV irradiation (λ = 365 nm) to induce the crosslinking process via photoinhibitor BP molecules finely distributed within PVP fibres. One of the probable scenarios of the photosensitisation process is the chemical reaction of photo excited radical generator (here BP) via hydrogen donor exchange with a solvent, a monomer or a polymer, which will be discussed in detail in further paragraphs [46]. Indeed, we observe the effects of this photocrosslinking process based on the obtained peak-distributions of fibre diameters, which decrease with the increase of the UV exposure time (Figure 1(h)). In particular, the average diameter of PVP (d_1_) and PVP-BP (d_2_) decreases by 27 nm ± 15 to d_3x_ = 240 ± 80 nm after 3 h crosslinking time (PVP-BP 3hUV) and further to d_3y_ = 180 ± 90 nm after 5 h (PVP-BP 5hUV). The addition of BP and the subsequent crosslinking process did not affect the smoothness of the obtained fibres, as confirmed by the atomic force microscopy amplitude error and phase images (Figure ESI 2). The crosslinking process can lead to formation of intermediate pyrrolidone hydroperoxide structure which can account for the efficient crosslinking, producing a sufficient level of macro radicals to form covalent bonds within fibres [47]. This in turn can lead to higher crowding of molecule, shortening the atomic distance between the BP molecules and PVP chains within fibres thereby decreasing the size of a given fibre. It is expected that this effect will scale as a function of crosslinking time until the reaction reaches plateau. These effects should directly impact the energy of bonds and intermolecular forces within fibres and thereby be directly correlated to Young’s modulus or Reduced Young’s Modulus (DMT) of fibres. Peak-force quantitative nanomechanical mapping (PF-QNM) was used to determine the changes in the DMT modulus of the obtained individual fibres prior to control PVP (Figure 1(i)) and after the crosslinking with BP (3 and 5 h UV irradiation) (Figure 1(j,k)). The average DMT modulus obtained for the control PVP fibres was 4.1 ± 0.8 GPa, the value in good agreement with that obtained by others [48]. We observed increase in the DMT modulus from non-cross-linked PVP control to cross-linked fibres: DMT modulus of PVP-BP 3hUV and PVP-BP 5hUV was similar: 7.2 ± 0.5 GPa and 5.9 ± 1.2 GPa, respectively, suggesting the possibility of BP crosslinking reaction. In particular, the obtained values imply that 3 h wasa sufficient enough crosslinking time for BP molecules to react (Figure ESI 3).

**Figure 2.**
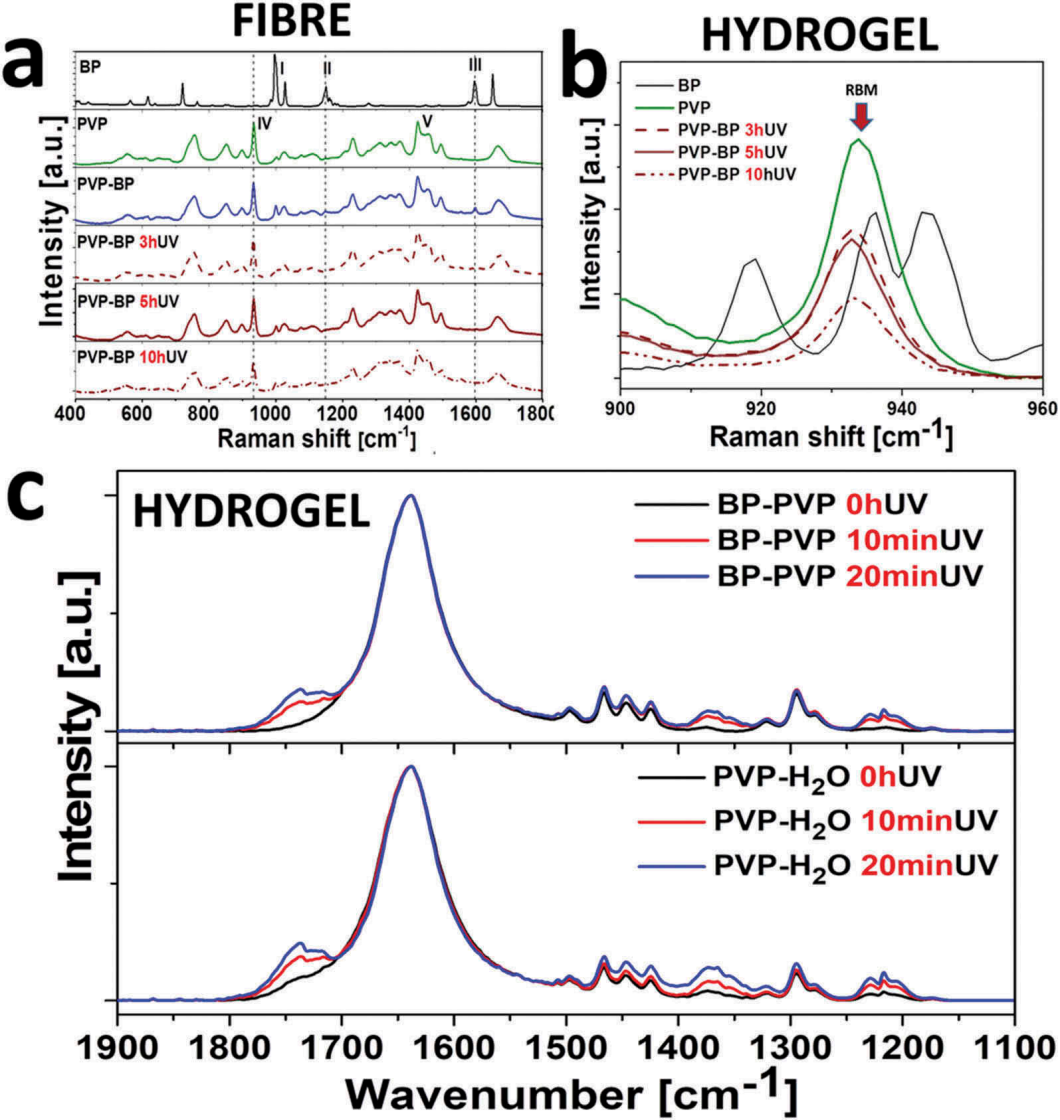
Raman spectra of 17 wt% PVP without and with 2 wt% BP, after 0, 3, 5 and 10 h of UV exposure (a). Selected region of 900–960 cm^−1^ shows the decrease of the ring breathing mode (RBM) peak at 935 cm^−1^ with the UV time exposure increase (b). Normalized (at 1634 cm^−1^) FTIR absorption spectra of obtained for the PVP (bottom) and PVP-BP (top) under short UV irradiation times (0, 10 and 20 min) (c).

BP and PVP have distinct Raman spectra as can be seen in Figure 2(a,b). It was therefore possible to monitor the incorporation and UV crosslinking reaction of BP (Figure 2(a)). Starting from BP spectrum, the intense lines at 997 cm^−1^ and 1027 cm^−1^ (I) are assigned as the deformation vibration and the C-C stretching vibration of the phenyl ring. Next, vibration at 1149 cm^−1^ (II) is the stretching one from C_A_-C-C_B_ between two phenyl rings. The mode at 1596 cm^−1^ (III) corresponds to the phenyl ring C-C stretching vibrations [45]. In case of mats made of pure PVP aqueous suspensions, the ring vibration was observed at 931 cm^−1^ (IV). The bands between 1420 and 1498 cm^−1^ (V) could be assigned to *ν*(CC) stretching vibrations and *ν*(CN) in-plane vibrations [49]. The examination of PVP-BP 3hUV, 5hUV and 10hUV via Raman spectroscopy revealed that the phenyl ring C-C stretching vibration (III) mode at 1596 cm^−1^ disappeared just after 3 h of crosslinking (arrow), again, indicating that 3 h of crosslinking time is sufficient for a significant number of BP molecules to react within fibres. Simultaneously other phenyl bands at 997 cm^−1^ and 1027 cm^−1^ (I) remain constant throughout this experiment indicating that the basic structure of phenyl rings remain unchanged. The strong band 931 cm^−1^ in all PVP-BP crosslinked samples indicates that the polymer backbone remains undamaged. Additionally, the reduction of the pyrrolidone ring breathing mode (RBM) by about 65% after 10 h of UV irradiation implied for partial conversion of pyrrolidone ring thus stiffening the fibre structure (Figure 2(b)), as previously evidenced by the PF-QNM fibre mapping. To fully understand the PVP crosslinking process in aqueous environment, we further performed the FTIR spectroscopic analysis of the PVP in H_2_O and PVP-BP, after 0, 10 and 20 min of UV irradiation. The short UV exposure time was selected to establish the initial mechanism of the crosslinking process. In case of both, PVP-BP and PVP-H_2_O, there are three main spectral regions which intensity increases with the UV exposure time. The first one (1800–1650 cm^−1^) corresponds to the symmetric stretching mode of the imide groups C = O (1768 cm^−1^) and asymmetric stretching mode of the two C = O groups in the succinimide ring (1698 cm^−1^) (Figure 2(c)). As a control, the same experiment involving UV exposure of PVP dissolved in 30% H_2_O_2_ for 0, 10, 20 min was conducted (Figure ESI 4). Additionally, the integrand field of the FTIR spectrum in the region between 1800 and 1650 cm^−1^ vs UV time exposure was estimated for all PVP-BP, PVP-H_2_O and PVP-H_2_O_2_ (Figure ESI 5). According to the data obtained after linear fitting, the crosslinking reaction rate was significantly higher for PVP-BP (slope = 5.1 ± 0.5; R^2^ = 0.98) and PVP-H_2_O_2_ (slope = 5.6 ± 0.5; R^2^ = 0.98) than for PVP-H_2_O (slope = 3.8 ± 0.9; R^2^ = 0.88).

**Figure 3.**
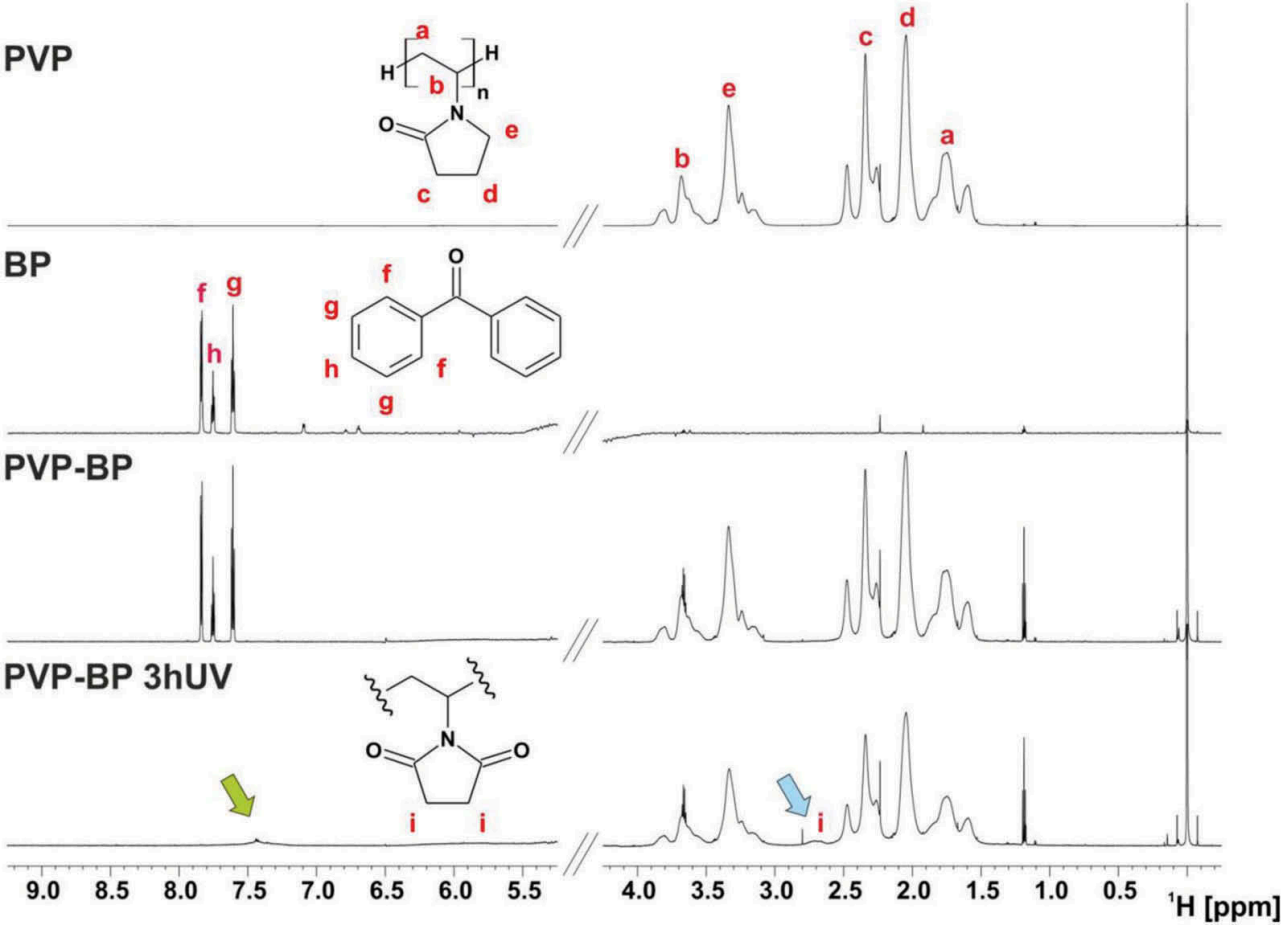
^1^H NMR spectra of PVP, BP and PVP-BP, before and after 3 h of UV exposure (800 MHz, 60% H_2_O/40% D_2_O, 25°C). Green arrow (~7.5 ppm) indicates up-shifted and broadened signal of BP after 3 h irradiation. Blue arrow denotes additional broad signal around 2.6 ppm assigned to the methylene protons of the succinimide ring.

The crosslinking mechanism of PVP-BP solutions under UV irradiation in our case seemed very similar to that suggested by Zhu et al [47]. Therefore, we examined the exact cross-linking mechanism using NMR spectroscopy (Figure 3). The ^1^H NMR spectrum for PVP-BP was observed to be the sum of individual spectra acquired for BP and PVP itself. This indicated that before the UV irradiation there were no chemical bonds occurring between the PVP monomers and BP molecules. On the other hand, significant changes were observed in the NMR spectrum of PVP-BP after 3 h of UV exposure (PVP-BP 3hUV), specifically: (i) signals around 7.5 ppm corresponding to BP were shifted and strongly broadened which resulted in their almost complete disappearance and (ii) appearance of an additional broad signal around 2.6 ppm. It was shown that the UV irradiation of aqueous solution of BP molecules leads to H_2_O_2_ production [50–52]. Additionally, Zhu et al. showed that irradiating the PVP and H_2_O_2_ suspension leads to the efficient crosslinking and production of sufficient level of macro radicals that lead to formation of stable hydrogels [47]. The final products of the PVP-BP after 3 h of UV cross-linking reaction include cross-links between methane carbon and hydroperoxy intermediate, cross-links from the hydroperoxide intermediate rings, hydroxylation on the methine carbon of the main chain and succinimide ring within PVP structure. The additional resonance (δ_H_ = 2.6 ppm) observed in the NMR spectrum (Figure 3) could therefore be assigned to the methylene protons of the succinimide ring. The ^1^H NMR assignments were confirmed on the basis of ^1^H-^13^C heteronuclear single quantum coherence (HSQC) experiments where correlation signal between ^1^H at 2.6 ppm and ^13^C at 30.9 ppm with ^13^C chemical shift values typical for the carbon atoms of methylene groups of succinimide rings were observed (Figure ESI 6). A signal at 2.6 ppm in the ^1^H NMR spectrum was also observed in case of UV cross-linking of PVP dissolved in 30% H_2_O_2_ which confirmed the same products formation as in the case of BP molecules formation (Figure ESI 7). Teixeira et al. have successfully used the same mechanism to cross-link the poly(ethylene oxide) (PEO) into soft hydrogel-like structures [52]. The ^1^H NMR results are consistent with the FTIR and Raman spectroscopies analysis and confirm that the crosslinking has indeed occurred.

**Figure 4.**
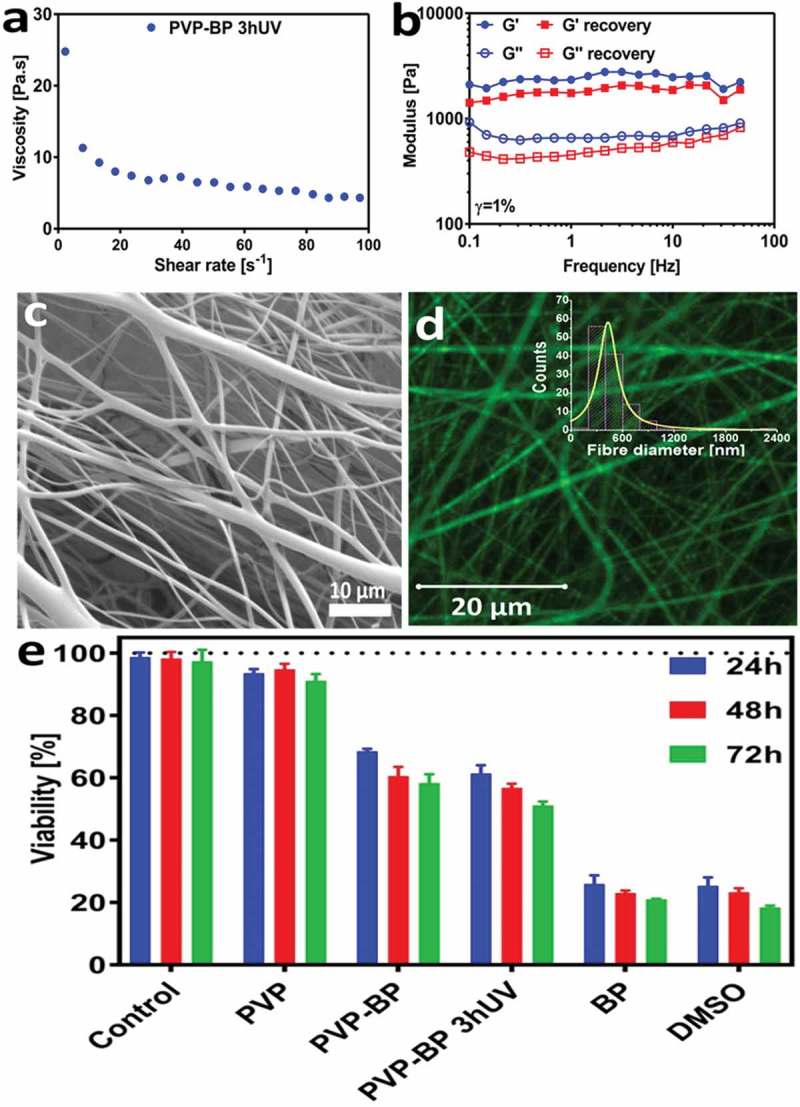
Viscosity as a function of shear rate (a), frequency sweep of the cross-linked PVP-BP 3hUV hydrogel prior to electrospinning (b). SEM micrographs of 17% PVP based fibre mat with 0.25% w/w lysozyme (c). Histogram of PVP-BP-LY fibre diameters. Fibre diameters were extracted from at least 200 fibres measured individually from a series of randomly chosen SEM images using ImageJ software (inset). Micrograph of PVP-BP-LY fibres (Rhodamine-B labelled) obtained using confocal microscopy (d). Viability of fibroblasts after 24, 48 and 72 h of incubation on electrospun PVP mat, PVP-BP and PVP-BP cross-linked for 3 h. The amount of BP used as a control in this experiment was equivalent to that used in the PVP-BP and PVP-BP 3hUV samples. Two controls were used: positive (10% dimethyl sulfoxide in DMEM) and negative (cells seeded in empty non-treated plastic wells containing DMEM (e)).

In order to increase the antibacterial properties of the produced mats, we aimed at encapsulating lysozyme: a fragile but very effective antibacterial enzyme. Lysozyme can be damaged by non-aqueous solutions [53] as well as direct and continuous UV light exposure [54]. Conveniently the PVP mats were already prepared in H_2_O, a perfect solvent for dissolving lysozyme molecules, yet we had to avoid direct UV light exposure during the BP crosslinking process. We produced PVP-BP dispersions using the same method as previously discussed, but we crosslinked it for 3 h **prior** to electrospinning to form a hydrogel and subsequently encapsulated lysozyme molecules within such produced hydrogel (PVP-BP-LY 3hUV). The rotational rheology confirmed that the viscosity of the cross-linked PVP-BP 3hUV hydrogel has significantly increased in comparison to non-crosslinked sample (Figure 4(a,b)). Nevertheless, it still exhibited strong shear-thinning behaviour over the full range of shear rates used and by increasing syringe flow rate to 5 ml/h during electrospinning as well as distance between electrodes to 8 cm, it was still possible to fabricate electrospun mats of the similar characteristics to before. Oscillatory rheology further suggested that the produced sample is a chemically crosslinked hydrogel with a typical Gꞌ>Gꞌꞌ characteristics for this type of polymeric hydrogels (Figure 4(b)) [47,55]. It is, however, interesting to note that our hydrogels had 10-fold increase in Gꞌ value in comparison to hydrogels produced from PVP via UV crosslinking with the addition of glycerol [55]. Secondary frequency sweep was performed immediately after the first one to examine the stability of hydrogel structure due to shear. We observed that the PVP-BP 3hUV hydrogel’s Gꞌ has decreased to 71% of its original value (at 1 rad/s), but the hydrogel structure and characteristics persisted, which meant that the sample after shear-disruption (such as minimal disruption caused by the mixing of concentrated lysozyme) could still be electrospun.

PVP-BP-LY 3hUV mats consisted of smooth fibres with an average diameter size of 470 ± 280 nm (Figure 4(c,d)). The examination via optical microscopy confirmed the successful incorporation of rhodamine B labelled lysozyme into all fibres and its homogeneous distribution throughout the PVP-BP-LY 3hUV polymeric mat (Figure 4(d)). These mats were subsequently tested for their antibacterial properties using AFM. We aimed to explore the possibility of using produced mats as antibacterial coatings/surfaces, and thereby their biocompatibility, by culturing fibroblasts over 3 days. Figure 4(e) indicates the viability of cells cultured on PVP, PVP-BP and PVP-BP 3hUV crosslinked fibres obtained via cell proliferation reagent WST-1. It is evident that pure PVP mats, which dissolved within minutes (as previously discussed), do not induce toxicity (cell viability > 90%) at all time points, similarly to other PVP fibrous systems reported before [56]. The fibroblast viability tests showed that the developed PVP-BP mats induce time-dependent manner weak cytotoxic effect until 1 day culture (65–70% of cell viability) and moderate cytotoxic activity (60–50% of cell viability) after 2 and 3 days of culture, whereas pure BP exhibits strong cytotoxicity (cell viability <25). PVP-BP mats before and after UV cross-linking induce toxicity toward fibroblasts at similar levels, while pure BP more than twice higher, suggesting that the incorporation of BP molecules within PVP fibres decreases the cytotoxicity of pure BPs. The toxicity and cancerogenicity of pure BP toward mammalian cells and bacteria, as well as in vivo studies, were previously described in literature. It is also worth noting that, incorporation of BP into PVP polymer fibres decrease cytotoxicity of BP reagent. These properties could in turn prove usefulness of the obtained mats as antibacterial dissolvable coatings to be used for sterilisation process of various medical equipment and surfaces that should be neither toxic nor irritating to people who handle it [57].

**Figure 5.**
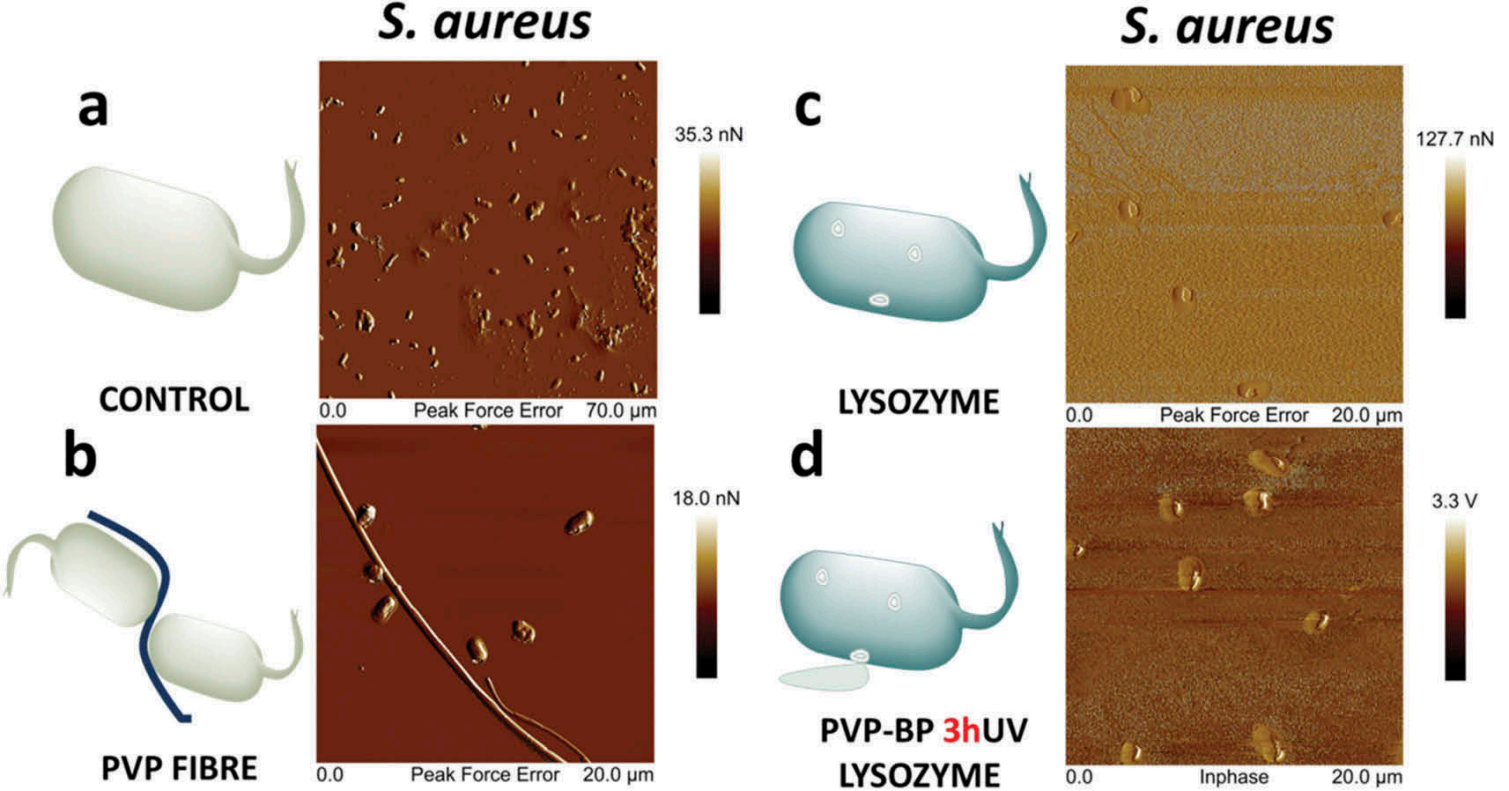
Atomic force microscopy images of bacteria cultured for 24 h alone (a), with PVP electrospun mat (b), lysozyme (c) and PVP-BP-LY 3hUV (d). Please note the dissimilar scales of the images.

Figure 5(a) shows an AFM image of *S. aureus* negative control sample. The AFM scan showed many adherent bacteria with undisrupted membranes. Closed peak force error (PKE) (Figure 5(a)) images show the two stacks of intact pairs of bacteria with a very smooth cell wall, same as in other reports [58]. We observed the high tendency of bacteria to aggregate around undissolved PVP fibres after their incubation with the pure PVP electrospun mat for 24 h (Figure 5(b)). Interestingly, bacteria surrounding the fibres had their cell-wall disrupted, indicating that PVP fibres alone may influence the survivability of *S. aureus*. After incubating *S. aureus* with pure LY we observed a large number of damaged bacteria, evidenced by their flat structure and a large rupture point, strongly visible in peak force error AFM image (Figure 5(c)). As already revealed in a number of reports [59], lysozyme can affect several Gram-positive and Gram-negative pathogens. It can be also clearly seen on these images that the surface of the substrate became rougher, suggesting adsorption of leaked cytoplasm and inner parts of bacteria onto it. Bacteria encapsulated with PVP-BP-LY 3hUV fibres showed very similar results to that of pure LY on an in-phase image (Figure 5(d)), which means that PVP-BP-LY 3hUV can be used as a delivery vehicle of LY for preventing bacteria growth. In general, after incubating bacteria with LY and PVP-BP-LY 3hUV the overall number of bacteria was lower than in the case of negative control and pure PVP, since more of bacteria were broken down during the 24 h incubation time. Since these mats will eventually dissolve in water and PVP will be bound to BP molecules, they could easily be wiped off with a wet wipe. It is possible that the previously observed induced moderate toxicity mechanism for fibroblast also plays a role against *S. aureus*. Nevertheless, the bacteria wall disruption by lysozyme (positive control) and PVP-BP-LY was clearly observed, suggesting that the main mechanism of *S. aureus* lysis is affected by hydrolysis of the peptidoglycan present in cell walls by lysozyme.

## 4. Conclusions

Using UV irradiation, we fabricated crosslinked PVP hydrogels in the presence of benzophenone molecules. All produced hydrogels were characterised in detail using spectroscopic methods. The additional resonances observed in the NMR spectra confirmed the presence of one of the reaction products which proved the crosslinking process. This was further confirmed via FTIR and Raman spectroscopy investigations of relevant chemical stretching and vibrational modes. This in-depth characterisation of the crosslinking process shed a new light into the possible facile chemical improvements that could be done to increase the integrity or mechanical properties of electrospun mats from hydrogels. We then used the electrospinning route to fabricate the selection of PVP polymer fibrous mats successfully functionalised with antibacterial lysozyme biomolecule. We confirmed the incorporation of rhodamine B labelled lysozyme into the fibre and its homogeneous distribution throughout the polymeric mat using optical microscopy. Furthermore, after *S. aureus* incubation with pure lysozyme, a significant number of damaged bacteria were observed with a flat structure and large rupture points caused by cytoplasm leakage. The obtained mats were found to not induce significant toxicity toward fibroblast cell line. We have finally observed the antibacterial properties of lysozyme encapsulated within PVP-BP 3hUV providing promising alternatives to existing antibacterial coatings for health-care applications. This observation provides evidence that incorporation of lysozyme biomolecules into PVP-BP fibres does not affect their biological functions (antibacterial properties).

## Supporting information

Graphs showing the SEM micrographs of mats fabricated from 13%, 15% and 17% of PVP water solution (Figure ESI 1); tapping mode AFM images obtained for PVP fibres, PVP-BP, PVP-BP 3hUV and PVP-BP 5hUV (Figure ESI 2); PF-QNM AFM images of individual PVP fibres (Figure ESI 3); FTIR absorption spectra for the PVP-H_2_O_2_ under UV irradiation times (0, 10 and 20 min) (Figure ESI 4); Integrand field of the FTIR absorption peak vs UV time and the linear fitting curves for PVP-H_2_O_2_, PVP-H_2_O, PVP-BP-H_2_O (Figure ESI 5); 2D NMR ^1^H-^13^C HSQC spectrum of PVP-BP (Figure ESI 6); ^1^H NMR spectra of the PVP cross-linking reaction with H_2_O_2_ (Figure ESI 7).
