# Supplementary material for: UV cross-linked polyvinylpyrrolidone electrospun fibres as antibacterial surfaces
Source: Sci Technol Adv Mater. 2019 Sep 17;20(1):979–91. doi: 10.1080/14686996.2019.1667737 (PMC6818115; doi:10.1080/14686996.2019.1667737)
Supplement: Supplemental Material [file TSTA_A_1667737_SM4181.pdf]

UV cross-linked polyvinylpyrrolidone electrospun fibres as antibacterial surfaces – Supporting Information

Barbara M. Maciejewska[1,*], Jacek K. Wychowaniec[1,♦], Marta Woźniak-Budych[1], Łukasz Popenda[1], Alicja Warowicka[1,4], Klaudia Golba[1], Jagoda Litowczenko[1,2], Zbigniew Fojud[3], Beata Wereszczyńska[1,3], Stefan Jurga[1]

[1]NanoBioMedical Centre, Adam Mickiewicz University, Umultowska 85, PL61614 Poznań, Poland

[2]Department of Molecular Virology, Faculty of Biology, Adam Mickiewicz University, Umultowska 89, PL61614 Poznań, Poland

[3]Department of Macromolecular Physics, Faculty of Physics, Adam Mickiewicz University, Umultowska 85, PL61614 Poznań, Poland

[4]Department of Animal Physiology and Development, Faculty of Biology, Adam Mickiewicz University, Umultowska 89, PL61614 Poznań, Poland

*Corresponding author: Barbara M. Maciejewska, e-mail: bmacieje@amu.edu.pl

Present Addresses

♦Current address: School of Chemistry, University College Dublin, Belfield, Dublin 4, Ireland, e-mail: jacek.wychowaniec@ucd.ie.

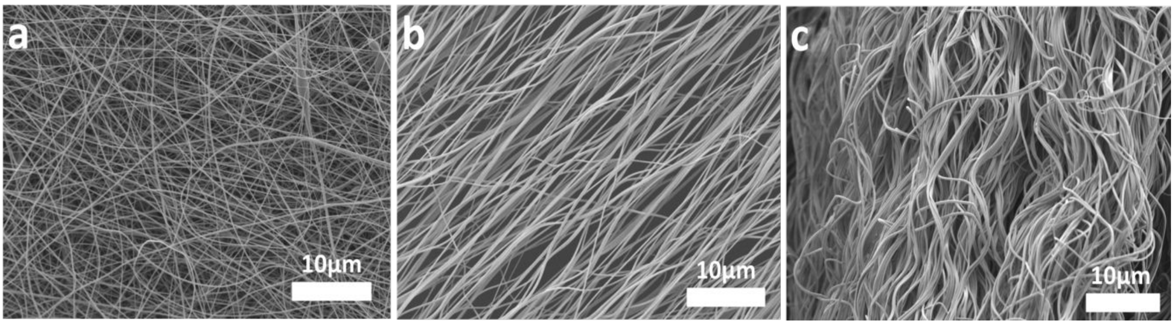

Figure ESI 1: *SEM micrographs of mats fabricated from (a) 13, (b) 15 and (c) 17 wt% of PVP water solution.*

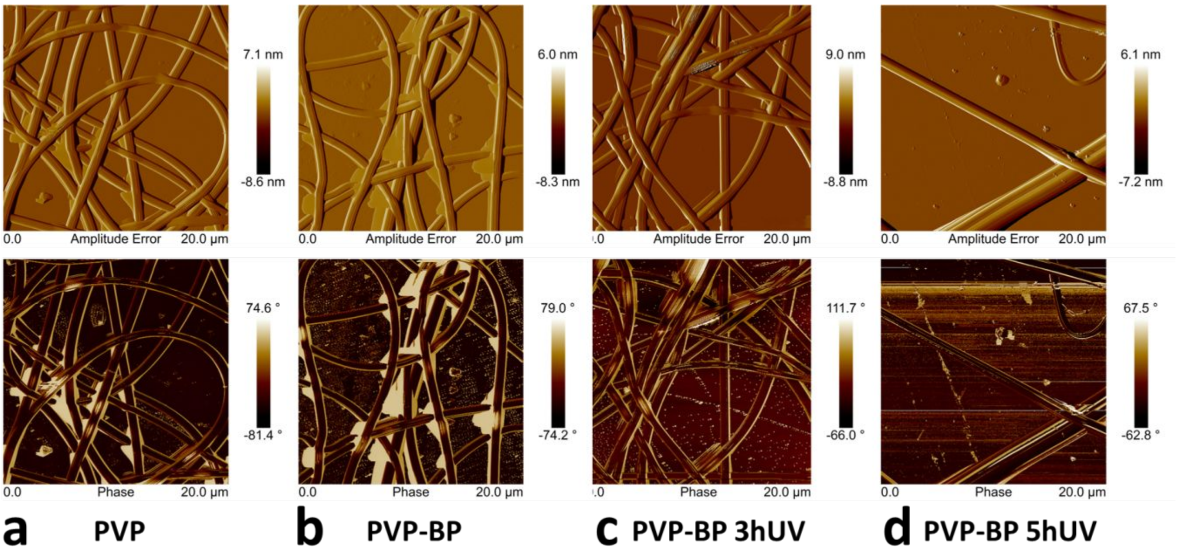

Figure ESI 2: *Tapping AFM images of obtained PVP fibers (a) control (b), with 2 wt% BP (c), 2 wt% BP after 3hs and (d), 5hs of UV exposure. Top row shows amplitude error images and bottom row phase images.*

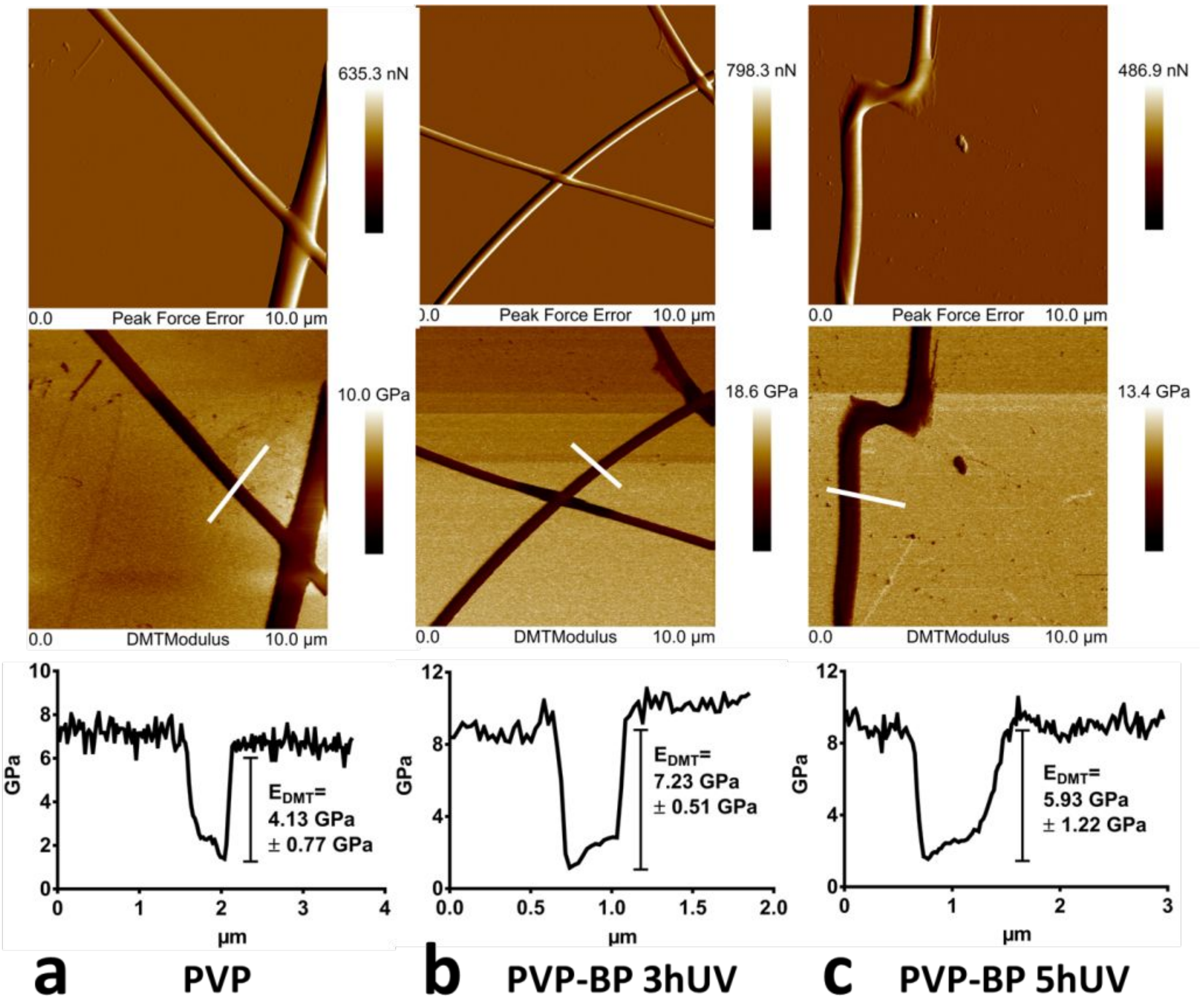

Figure ESI 3: *PF-QNM AFM images of individual PVP fibres (a) control (b), 2% w/w BP after 3hs and (c), 5hs of UV exposure. Top row shows peak force error images, middle row shows DMT Modulus images and bottom row shows exemplar cross-sectional measurements of DMT modulus at the places where white line was drawn on the DMT modulus images. The average values presented in the graph (± SD) are taken over the selection of all points along at least three fibres (n = 3) and referenced to the value of Si substrate, here taken as 0.*

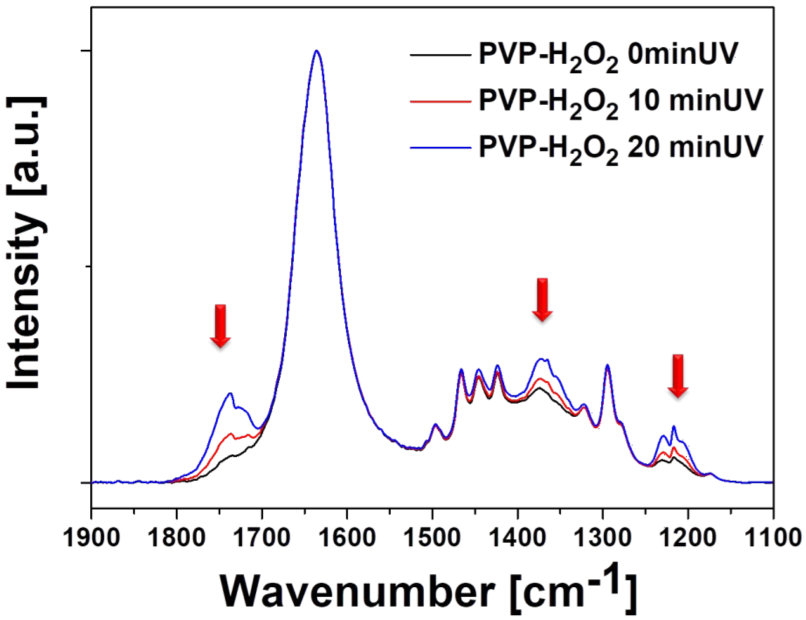

Figure ESI 4: *Normalized (to 1634 cm*$^{-1}$*) FTIR absorption spectra obtained for the PVP-*H_2O_2 *under short UV irradiation times (10 and 20 minutes).*

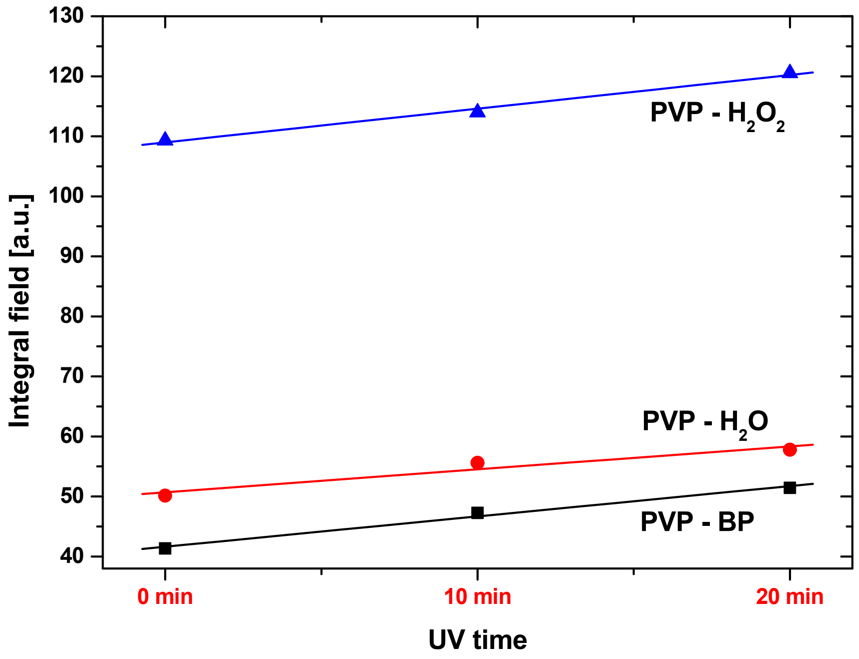

Figure ESI 5: *The integral field of the FTIR absorption peak (between 1800-1650cm*$^{-1}$*) vs UV time and the linear fitting curves for PVP-*H_2O_2*, PVP-*H_2O*, PVP-BP-*H_2O*.*

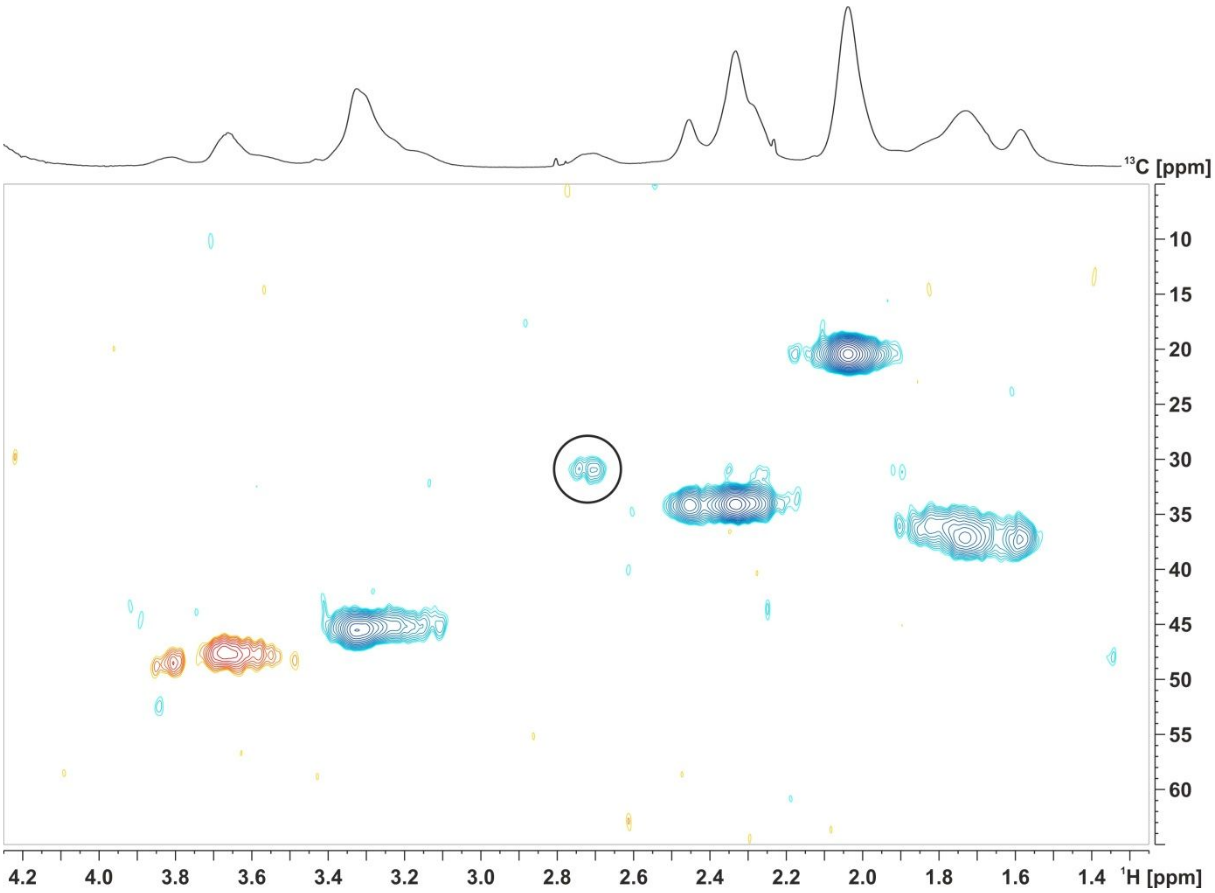

Figure ESI 6: *^{1}H-^{13}C HSQC spectrum of PVP-BP aqueous solution (H_2O) after 3hs of UV irradiation acquired at 55 °C. Circled cross-peak corresponds to coupling interactions between directly bounded ^{1}H and ^{13}C nuclei in succinimide ring.*

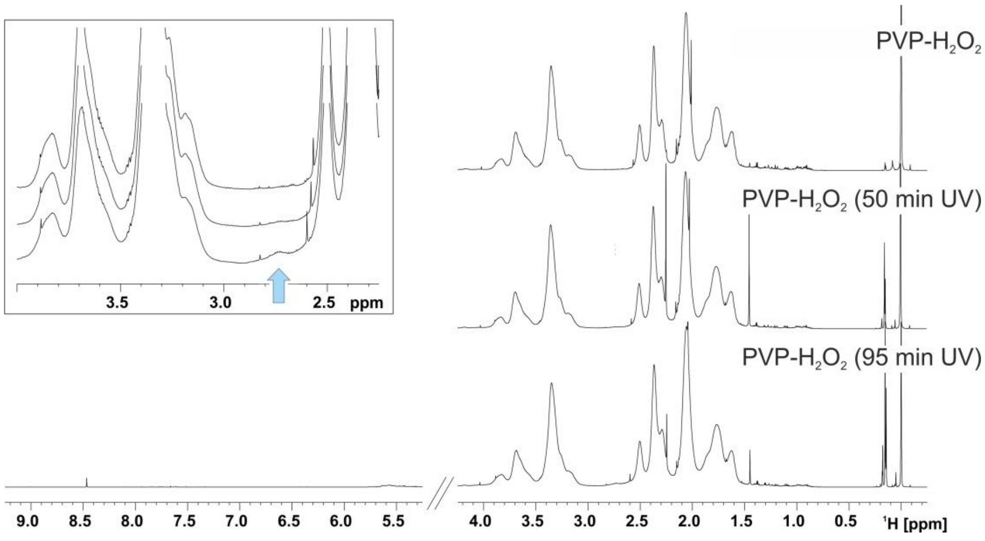

***Figure ESI** 7*: *^{1}H NMR spectra of PVP-H_2O_2 without any UV exposure and after 50 and 95 min of UV exposure (800 MHz, 60 vol% H_2O/40 vol% D_2O, 25 °C). Blue arrow denotes additional broad signal around 2.6 ppm assigned to the methylene protons of the succinimide ring.*
